# An alien intermediate host snail, *Radix rubiginosa* (Gastropoda: Lymnaeidae), on Unguja Island, Zanzibar

**DOI:** 10.1016/j.ijppaw.2026.101237

**Published:** 2026-05-08

**Authors:** Alexandra Juhász, Mtumweni Ali, Sam Jones, Carmen Ngo, Paula Cambero Guerra, E. James LaCourse, Othman Juma, Shaali M. Ame, J. Russell Stothard

**Affiliations:** a Institute of Medical Microbiology, Semmelweis University, Budapest, H-1089, Hungary; b Department of Tropical Disease Biology, Liverpool School of Tropical Medicine, Liverpool, L3 5QA, UK; c Neglected Tropical Diseases Programme, Zanzibar Ministry of Health, Zanzibar, Tanzania; d Zanzibar Livestock Research Institute, Ministry of Agriculture, Irrigation, Natural Resources and Livestock, Zanzibar, Tanzania

**Keywords:** Africa, Fascioliasis, Invasive species, *Radix natalensis*, *Radix rubiginosa*, Tanzania

## Abstract

In June 2025, two unusual populations of lymnaeid snails were encountered within peri-urban freshwater habitats of Unguja Island, Zanzibar (United Republic of Tanzania). These were noted within a wider malacological survey for freshwater intermediate host snails across twenty-one locations. Using an integrative taxonomic approach combining conchology and anatomy (n = 21) with mitochondrial 16S rDNA (n = 8) and mitochondrial *cox*1 (n = 3) sequence information, these lymnaeids were identified as *Radix rubiginosa* (Michelin, 1831). This is a first observation of this Southeast Asian alien species in East Africa, adding to its prior reports in South Africa. While no intrapopulation DNA sequence diversity was detected, very high DNA sequence similarities [16S rDNA(>99.4%) and *cox*1 (>99.6%] were noted, foremost with a Southeast Asian aquarium population. Upon field collection and then later by laboratory inspections (n=150), no trematode larvae were observed either upon cercarial shedding or by dissections. Furthermore, *ad hoc* parasitological screening of faeces from local cattle (n = 10) yielded no trematode eggs. Nonetheless, we raise a new regional surveillance flag for snail-borne diseases potentially transmissible by this alien intermediate host in Zanzibar.

## Highlights

• First detection of *Radix rubiginosa* in East Africa on Unguja Island, Tanzania.
• Identification corroborated by mitochondrial 16S rDNA and *cox*1 sequence analyses.
• High DNA similarities with Southeast Asian lineages suggest a recent introduction.
• A tangible risk of altered trematode transmission locally is newly flagged.

## Graphical abstract

**Figure:**
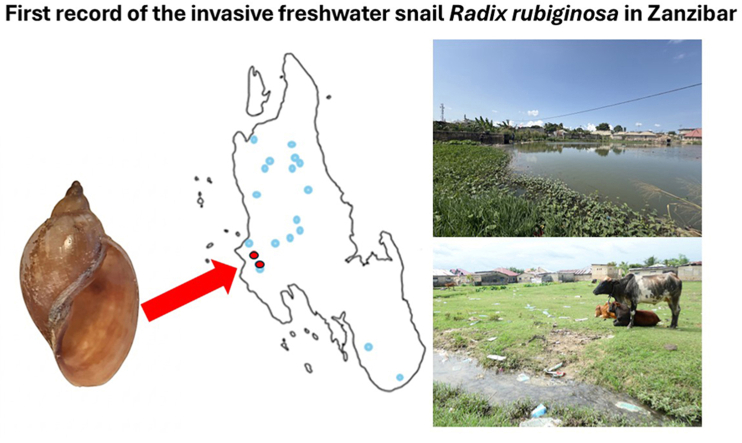

## 1 Introduction

Though numerous taxonomic controversies within the freshwater gastropod family Lymnaeidae remain (Correa et al., 2010; Aksenova et al., 2018), several snail species are globally recognized as keystone intermediate hosts for transmission of digenean trematodes of medical and veterinary importance (Bunnag et al., 1983; Srihakim and Pholpark, 1991; Charoenchai et al., 1997; Japa et al., 2021). As highlighted by Vinarski and Vázquez (2023), their wide distribution, ecological adaptability, and close association with parasite transmission, grant them special attention within freshwater ecology. Considering the radicines throughout Southeast Asia (Hoang Quang et al., 2024; Martviset et al., 2024), *Radix rubiginosa* (Michelin, 1831) is a central intermediate host of animal fascioliasis, as principally caused by *Fasciola gigantica* (Cobbold, 1855). A rather ubiquitous snail species, it occurs within a variety of natural and manmade habitats (Bunnag et al., 1983; Martviset et al., 2024), having been implicated in transmission of the veterinary schistosome *Schistosoma incognitum* Chandler 1926, alongside avian schistosomes of the genus *Trichobilharzia* (Skrjabin and Zakharov, 1920)(Japa et al., 2021). Of relevance here, *R. rubiginosa* was first noted in 2004 in South Africa within a tropical aquaculture facility in KwaZulu-Natal Province, with an informative morphological identification key provided to later assist in differentiation of it from *Radix natalensis* (Krauss, 1848), *Pseudosuccinea columella* (Say, 1817) and *Galba truncatula* (Müller, 1774) (Appleton and Miranda, 2015). In South Africa, *R. rubiginosa* is an invasive species and classified as a “hothouse alien” which has also been observed in the UK and Israel, reflecting its ability to establish in controlled environments and potentially outcompete native snails (Mienis, 1986; Anderson, 2005). Combined with its high reproductive capacity, broad environmental tolerance, and role as an intermediate host for multiple trematodes, this snail species makes it a tangible threat to freshwater biodiversity and public health if introduced into new aquatic ecosystems (Appleton and Miranda, 2015). Nonetheless, natural infections with *F. gigantica* have yet to be demonstrated within this alien snail species (Nyagura et al., 2022).

On the coastal East African islands of Tanzania, there has been a long history of snail-borne disease surveillance with various medical malacological studies, having greatest focus upon *Bulinus globosus* (Morelet, 1866) due to its major involvement in transmission of urogenital schistosomiasis (Muhsin et al., 2022; Trippler et al., 2023). In relation to the lymnaeids on Zanzibar, *R*. *natalensis* is commonly reported on Pemba (Trippler et al., 2024) but can be considered absent, or very rare, on Unguja (Brown, 1994). As part of ongoing island-wide freshwater snail surveys, primarily targeting intermediate hosts of urogenital schistosomiasis, our study reports on the first detection of *R. rubiginosa* on Unguja Island.

## 2 Methods

In June 2025, a routine island-wide freshwater malacological survey was conducted on Unguja, where a total of 19 sites and 2 sub-sites were surveyed (see the Online Appendix). Site selection was based on a combination of novel satellite imagery obtained via Google Earth Pro (version 7.3), previous malacological and pilot parasitological surveys for human or bovine schistosomiasis (Stothard and Rollinson, 1997; Ame et al., 2025). Of special note here, the presence of a lymnaeid, in high populational densities, came to the authors’ attention, after local consultation with Ministry of Health personnel who had recently undertaken a practical training demonstration in the use of the chemical molluscicide Bayluscide® (Muhsin et al., 2022).

At each of the 21 sites, two experienced researchers conducted snail collections, 15 min/site, with a focus on *Bulinus* species due to their medical relevance on Unguja. Water chemistry parameters including pH, conductivity (uS), total dissolved solids (ppm), and temperature (^o^C) were recorded using two handheld Hanna Instruments Pocket EC/TDS and pH testers, with each measurement repeated twice and averaged. Sites that were revisited for additional snail collections had an additional water chemistry measurement to monitor potential changes (see the Online Appendix).

**Fig. 1:**
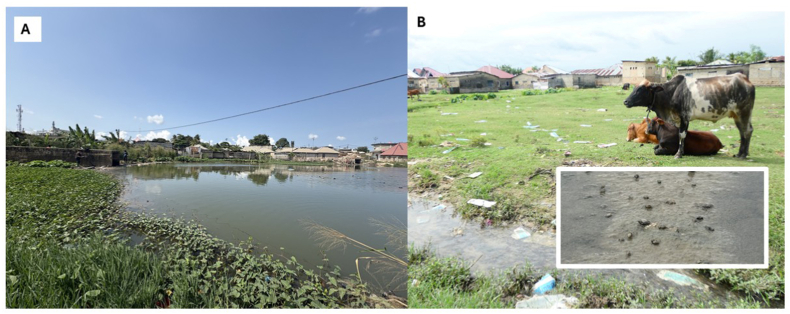
A) Sites photos of the locations where *R. rubiginosa* was first encountered, Mwantenga (A) runs on the same water source as Tomondo (B). Numerous snails were found on mud [small inset].

The unusual lymnaeids were only encountered at Mwantenga (S 6.172338°, E 39.223168°) (Fig. 1A) which is hydrologically connected to Tomondo (S 6.19439°, E 39.23165°), where free grazing cattle were also noted (Fig. 1B). Conducting an *ad hoc* parasitological inspection of these cattle, individual faecal samples were collected from ten animals and examined using both miracidia hatching tests, to detect schistosomes, and sedimentation techniques, to detect liver flukes, following standard parasitological protocols (Juhász et al., 2024a). Across the two sites, a total of 150 lymnaeid snails were collected by hand, whilst noting snails being more plentiful on exposed moist mud surface that often proved difficult to access by foot.

### 2.1 Cercarial shedding and snail characterization

All field-caught snails were initially screened for natural trematode infections through cercarial shedding by placing individuals in containers with mineral water and exposing them to light. After cercarial inspections, a representative selection of thirty snails was preserved in absolute ethanol and subsequently transported to the laboratory for conchological and anatomical assessments. A total of five snails were dissected; a soft body was extracted from the shell with hooked metal forceps. The empty shells were cleaned, dried and preserved at room temperature for shell morphometrics.

**Fig. 2:**
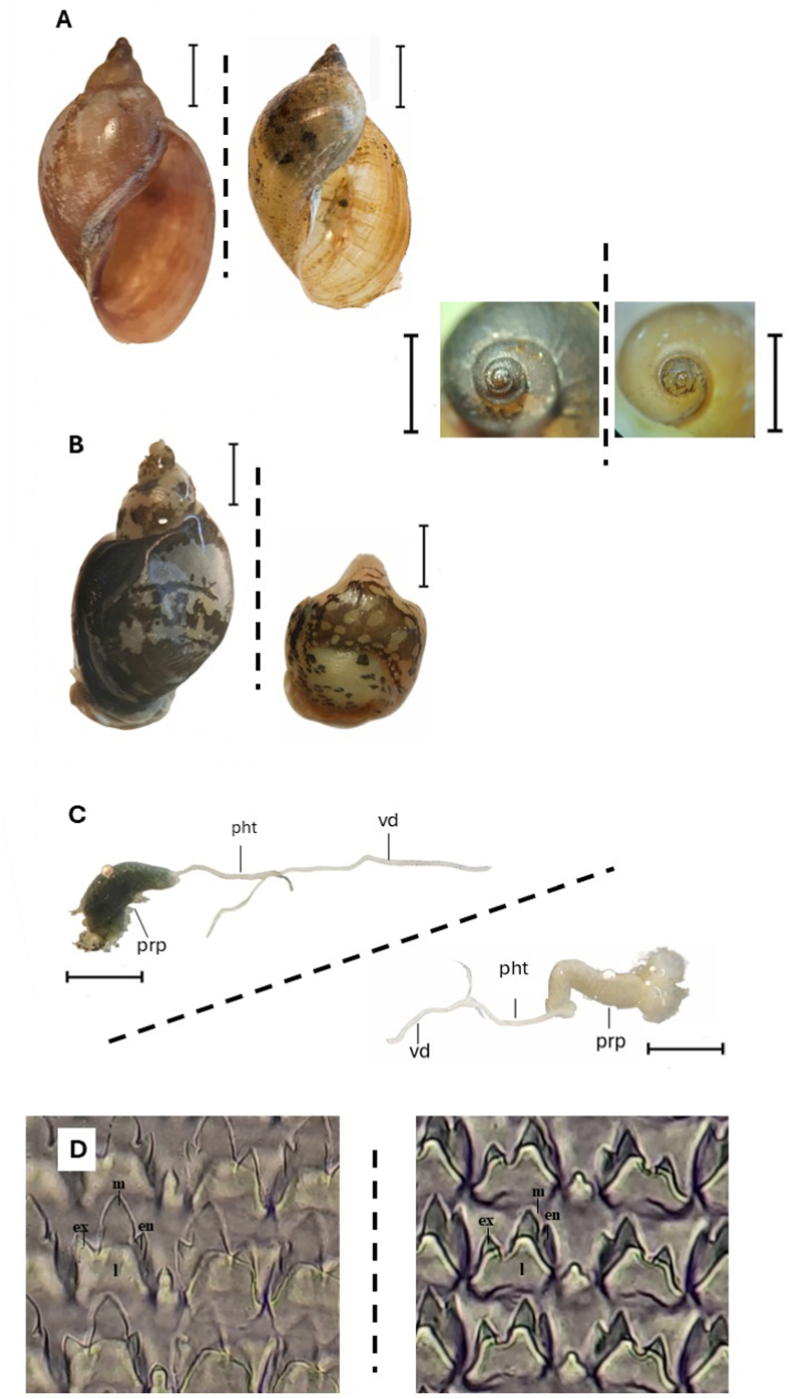
Morphological and anatomical comparison of *Radix rubiginosa* (left) and *Lymnaea natalensis* (right); dashed line indicates separation between species in each panel. A) Shell and protoconch morphology; B) mantle pigmentation; C) male reproductive system (pht = phallotheca, prp = praeputium, vd = vas deferens); D) radula. l = first lateral tooth, en = endocone, ex = exocone, m = mesocone). Scale bars: A,B = 3 mm; C = 2 mm.

Under the stereomicroscope, all snails were fully developed adults, according to the maturity of their genital organs. The male genital structure was dissected out of the soft body and measured using a stereo microscope (Fig. 2C). The buccal mass was dissected out and digested in lactic acid (≥88%) (Santa Cruz Biotechnology) for 24 h to free the radula from snail tissues and then examined under the compound light microscope (Fig. 2D). A total evidence approach was taken to assess each character against the current lymnaeid literature, as species-specific morphological keys remain tentative (Hubendick, 1951; Nadasan, 2011; Appleton and Miranda, 2015; Dumidae et al., 2024), with these specimens directly compared against two recently collected *R. natalensis* snails preserved in absolute ethanol from Matta Barrage, Cameroon.

### 2.2 Characterisation of mitochondrial 16S rDNA and cox1

For species identification, genomic DNA was extracted from the foot muscle of 21 specimens, preserved in absolute ethanol, using the hexadecyltrimethylammonium bromide (CTAB) method, followed by phenol-chloroform extraction and ethanol precipitation (Kaset et al., 2010). Molecular taxonomy first focused on the well-known mitochondrial marker, the large subunit 16S ribosomal DNA which has been a useful region for taxonomic studies since the pioneering work of Remigio and Blair (1997). A partial region of the 16S rDNA region was amplified using the primers 16brm (5′-CCGGTCTGAACTCTGATCAT-3′) and 16arm (5′-CGCCTGTTTATCAAAAACAT-3′). PCR products were visualized on 1.5% agarose gels, and positive amplicons were purified and subjected to bidirectional Sanger sequencing, a total of eight successful amplicons were sent for sequencing obtaining a 346 bp consensus as determined in both directions. Since there was no variation between samples, a single sequence was deposited in GenBank (Accession Number: PZ154509). A second mitochondrial locus was targeted, a 607 bp sub-region of the mitochondrial cytochrome oxidase sub-unit 1 (*cox*1) gene was amplified using the primers LCO1490 (5′-GGTCAACAAATCATAAAGATATTGG-3′) and HCO2198 (5′-TAAACTTCAGGGTGACCAAAAAATCA-3′), from three snails. Since there was no variation between samples, a single *cox*1 sequence was deposited in GenBank (Accession Number: PZ343134).

## 3 Results

The two sites where lymnaeid snails were encountered, Mwantenga and Tomondo, are peri-urban freshwater habitats on Unguja Island that are hydrologically connected within a low-lying drainage system influenced by seasonal flooding. No other inspected sites on Unguja harboured lymnaeids. These two sites were characterised by shallow, slow-moving or stagnant water bodies with extensive exposed muddy margins. Water chemistry measurements (see Online Appendix) indicated broadly comparable environmental conditions across sites, with circumneutral to slightly alkaline pH, moderate conductivity, and elevated total dissolved solids, consistent with organically enriched freshwater habitats subject to anthropogenic and livestock-associated inputs.

At each of these two locations, lymnaeid snails were collected, with individuals most frequently observed on exposed moist mud rather than on fully submerged substrates. This ecological trait was notable, as it contrasts with typical observations of *R*. *natalensis* in the region, which is more commonly associated with shallow water rather than emergent muddy surfaces. All field-caught snails were examined for trematode infections by cercarial shedding and a sub-set by anatomical dissection. No evidence of trematode infection was detected; all dissected individuals were negative. Furthermore, all bovine faecal samples collected from sites where these snails were detected tested negative for trematode infection by either miracidia hatching or by sedimentation assays.

Following the identification key of Appleton and Miranda (2015), morphologically, the most informative discriminant conchological characters were shell length, the length of the last body whorl, and aperture width. These parameters provided suitable applicable criteria for first distinguishing *R*. *rubiginosa* from *R*. *natalensis* (Fig. 2A). *Radix rubiginosa* consistently exhibited larger, more broadly elongate shells with proportionally stretched body whorls, whereas *R. natalensis* has smaller, more ovate shells with comparatively shorter body whorls and has a more fragile shell, more easily mechanically damaged. In addition, *R. rubiginosa* possessed a relatively narrower aperture compared to the broader apertural morphology, for example, as observed in *R. natalensis* specimens from Cameroon. In *R. rubiginosa*, the slightly greater spire height is attributable to an additional shell whorl (Fig. 2A).

In contrast, reproductive anatomy showed a high degree of similarity between the two taxa, with limited interspecific variation (Fig. 2C). However, some subtle radular morphology differences were observed (Fig. 2D) and may serve as an additional diagnostic feature when further material is examined. In *R. rubiginosa*, structures of the first lateral tooth revealed a symmetrical arrangement of the endocone and exocone, with a highly prominent mesocone, whereas, in *R. natalensis*, the endocone and exocone of the first lateral tooth were markedly asymmetrical. Mantle pigmentation provided a clear additional distinguishing feature, *R. rubiginosa* displayed a distinctive mottled dark mantle with irregular pale patches, whereas *R. natalensis* exhibited a more uniformly spotted pigmentation pattern (Fig. 2B).

Upon BLAST analysis of our 16S rDNA sequence PZ154509 revealed no exact matches on GenBank. However, a 99.4% sequence similarity was noted to *R*. *rubiginosa* isolate ALA2 16S rDNA gene (Accession Number KU318356.1) from Singapore, as reported by Ng et al. (2016) in their survey of invasive aquatic molluscs within the ornamental pet trade. Several additional matches were identified, principally with other *R. rubiginosa* isolates from Thailand, China and Malaysia. A summary alignment of variable nucleotides within PZ1151509 and KU318356.1 is shown with the equivalent region for *R*. *natalensis* (JN794317.1) from Lake Malawi, Malawi, having 89.3% sequence identity (Table 1). BLAST analysis of the *cox*1 sequence PZ343134 showed a 99.7% sequence similarity to *R. rubiginosa* with the same isolate ALA2 (Accession Number KU318330.1) with additional high-similarity matches identified from isolates in Malaysia, the Philippines and Vietnam. Again, a summary of variable nucleotides between our Ungujan isolate PZ343134, KU318330.1 and a *R. natalensis* (PP528802.1) from Kigali, Rwanda with a 87.8% sequence similarity is shown (Table 2).

**Table 1:** Variable nucleotide positions in 16S rDNA from Ungujan *R. rubiginosa* (PZ154509.1) compared with the closest match *R. rubiginosa* (KU318356.1) from Singapore and *Radix natalensis* (JN794317.1) from Lake Malawi, Malawi. Numbered positions correspond to alignment of base pairs from PZ154509.1.

Variable Nucleotide Positions (bp)										
Taxon (GenBank Accession)	8	34	55	56	60	61	73	81	97	99
*Radix rubiginosa* (PZ154509)	G	T	T	C	A	A	T	G	A	T
*Radix rubiginosa* (KU318356.1)	T	T	T	C	A	A	T	G	A	T
*Radix natalensis* (JN794317.1)	T	A	C	T	G	G	C	T	T	A

100	101	102	125	140	143	146	148	152	153
T	A	A	T	C	C	T	T	A	G
T	A	A	T	C	C	T	T	A	G
C	T	T	A	T	T	A	A	G	A

196	197	199	202	206	207	216	217	228	256
A	A	A	C	T	A	G	T	A	G
A	A	A	C	T	G	G	T	A	G
T	-	T	A	A	T	A	G	T	A

261	262	267	270	297	298	314	336	337	338
T	T	T	G	T	T	T	T	T	A
T	T	T	G	T	T	T	T	T	A
A	A	C	A	A	A	A	A	A	T

**Table 2:** Variable nucleotide *cox*1 positions in Ungujan *R. rubiginosa* (PZ343134) compared with the closest match *R. rubiginosa* (KU318330.1) from Singapore and *Radix natalensis* (PP528802.1) from Kigali, Rwanda. Positions correspond to alignment base pair positions from PZ154509.1.

Variable Nucleotide Positions (bp)																					
Taxon (GenBank Accession)	2	6	8	26	31	41	44	47	50	53	56	65	77	79	80	91	95	98	113	122	128
*Radix rubiginosa* (PZ343134)	A	C	T	T	A	C	T	T	A	T	A	G	T	G	A	C	A	A	A	C	T
*Radix rubiginosa* (KU318330.1)	A	C	T	T	A	A	A	T	A	T	A	G	T	G	A	C	A	A	A	C	T
*Radix natalensis* (PP528802.1)	G	G	A	A	G	A	A	A	T	C	T	A	C	A	G	T	T	T	G	T	G

134	137	140	146	149	161	167	179	191	197	206	224	226	227	242	248	257	260	265	266
A	T	A	T	G	A	A	T	T	T	G	C	G	A	T	G	G	C	G	T
A	T	A	T	G	A	A	T	T	T	G	C	G	A	T	G	G	C	G	T
T	C	G	C	A	T	G	A	A	C	A	T	A	G	A	A	C	T	A	A

275	284	290	296	299	302	311	314	317	326	328	331	353	389	407	425	443	479	485	491
T	A	T	C	T	T	T	G	A	T	A	T	G	C	A	A	A	A	T	C
T	A	T	C	T	T	T	G	A	T	A	T	G	C	A	A	A	A	T	C
A	T	A	T	A	C	A	T	G	A	G	C	A	A	T	G	T	G	A	T

506	521	522	523	530	539	541	542	550	553	557	563	569	578	587
T	A	G	A	C	T	G	T	A	A	T	A	T	G	A
T	A	G	A	C	T	G	T	A	A	T	A	T	G	A
C	T	A	T	T	C	A	C	G	G	C	T	C	A	T

## 4 Discussion

Our current study contributes to a growing body of evidence that certain freshwater lymnaeid snails are colonising Africa from presumably intercontinental origins, e.g. *Orientogalba viridis* (Quoy and Gaimard, 1832) and *Pseudosuccinea columella* (Say, 1817), which have tangible implications for emerging snail-borne parasitic diseases (Jones et al., 2024; Juhász et al., 2024b). To the best of our knowledge, we have confirmed the novel occurrence of *R. rubiginosa* on Unguja Island. This substantively expands the known distribution of this lymnaeid snail within Africa and represents a first record in East Africa. Previous reports of this species on the African continent have been restricted to South Africa, where it has been considered a recently introduced and potentially invasive freshwater snail (Appleton and Miranda, 2015). Its detection on Unguja therefore raises important ecological and epidemiological considerations regarding both introduction pathways and possible impacts on local snail-borne trematodes (Pennance et al., 2022).

We identified *R. rubiginosa* upon a combined morphological and molecular approach, being mindful of the well-recognized taxonomic complexity of the family Lymnaeidae. Morphological identification alone is often insufficient due to the high phenotypic plasticity of shell characters in lymnaeid snails, which can vary substantially depending on environmental conditions. In this instance, two unusual features first brought this snail to our attention placing it at odds with characteristics of the more well regionally-known *R. natalensis*. First, it was found in plentiful numbers in predominantly amphibious habitats on moist mud. Second, the shell spire was more prominent, with a more rustic colour overall, being more robust to physical damage.

To confirm the alien nature of this snail, molecular taxonomic characters using mitochondrial 16S rDNA, and *cox*1, were foundational for incriminating a more reliable species identification, distinguishing it from other closely resembling radicine taxa. Indeed, while our Ungujan sequences were unique, the very high genetic similarities observed between these specimens and the aquarium isolate from Singapore and further Southeast Asian lineages supports the hypothesis of a recent alien introduction. However, since the *R. rubiginosa*'s 16S rDNA sequences reported in Appleton and Miranda (2015) were not available for comparison, we cannot unequivocally judge if our Ungujan lineage is identical to these South African populations, first noted in 2004. Clearly, using 16S rDNA and *cox*1 sequence evidence confirms a non-native African lineage with a probable Southeast Asian origin with recent populational ancestry (Ng et al., 2016). Nevertheless, this snail's occurrence on Unguja is likely linked to anthropogenic activities such as aquaculture, aquatic plant trade, or inadvertent transport with agricultural materials, as highlighted elsewhere by Ng et al. (2016) in their treatise on freshwater molluscs within the ornamental aquatic pet trade. Looking beyond Unguja, the medium-term outlook for *R. rubiginosa* in the Western Indian Ocean region warrants consideration. Although currently not formally documented in the published literature, it is reportedly now widespread on Mauritius. This raises the possibility of either multiple independent introductions or an ongoing, as of yet under-detected, regional spread facilitated by human activities and inter-island connectivity. Consequently, targeted malacological surveys across neighbouring islands may reveal additional populations, highlighting the need for enhanced regional surveillance and biosecurity awareness for this non-indigenous species. After gaining first foothold on Unguja, its further dispersal may be facilitated by flooding events between the two sampled locations that have been reported locally, enhancing passive dispersal mechanisms (Nassor and Makame, 2021), notwithstanding other carriage movements on either mammals and/or birds (Boag, 1986; Figuerola and Green, 2002; Juhász and Majoros, 2023), foraging along this local watercourse. It is open to speculation if, in coming years, this snail species will be found within the other 19 Ungujan habitats where it was proven absent.

The potential epidemiological implications of the permanent establishment of *R. rubiginosa* in Zanzibar warrant special attention, as this might be a future seeding location for translocation(s) onto nearby mainland East Africa. In its native Southeast Asian range, this species serves as an intermediate host for several trematodiases of medical and veterinary importance, including fascioliasis and avian schistosomiasis. Zanzibar already hosts extensive freshwater ecosystems where livestock, wildlife, and human populations interact with aquatic environments, creating opportunities for the establishment of new parasite transmission cycles if competent intermediate snail hosts become established. The introduction of a new lymnaeid species could therefore alter the local ecology of parasitic trematodes through mechanisms such as host switching, parasite amplification, or competition with existing permissive snail hosts (Lockyer et al., 2003).

Importantly, the potential ecological interactions between *R. rubiginosa* and the native lymnaeid fauna in Zanzibar remain uncertain. Notably, *R. natalensis* has not been observed or collected on Unguja for approximately two decades, suggesting that any direct competition between these two snail species is currently unlikely. Rather, the present finding may represent the introduction of an non-indigenous lymnaeid into an available ecological niche. However, interactions with other freshwater snail species, natural or introduced, such as *Physa acuta* (Albrecht et al., 2025), should not be overlooked. Future scenarios may include niche overlap or competitive displacement, highlighting the need for closer, long-term ecological monitoring to better understand the establishment dynamics and potential community-level impacts of this alien snail species.

## 5 Conclusion

Despite several limitations, in that we have yet to confirm the long-term presence of this snail species on Unguja and determine its natural or experimental capacity for transmission of local trematodes, we have evidenced the importance of routine malacological surveillance. Foremost, early detection of an invasive alien snail species is critical for understanding any future changes in the transmission of local trematodes to better inform appropriate risk mitigation and appropriate environmental management.

## Ethical approval

Snail-borne disease surveillance is within a governmental mandate of the Zanzibar Ministry of Health. The authors assert that all procedures contributing to this work comply with the ethical standards of the relevant national and institutional guides. Experimentation was performed on invertebrate animals alone. The study was approved in the UK by the Research Ethics Committee of the Liverpool School of Tropical Medicine (LSTM). All cattle sampling used non-invasive faecal collection methods, and protocols were approved by the animal ethics committee of the Zanzibar Livestock Research Institute.

## Funding

This work forms part of the MSc research dissertations of Carmen Ngo. This work received no specific grant from any funding agency, commercial or not-for-profit sectors. Alexandra Juhász and Sam Jones receive salary support from the 10.13039/100010269Wellcome Trust and, in part, by the 10.13039/501100000272National Institute for Health Research (10.13039/501100000272NIHR) (using the UK's Official Development Assistance (ODA) Funding) and 10.13039/100010269Wellcome Trust [220818/Z/20/Z] under the NIHR-10.13039/100004440Wellcome Partnership for 10.13039/100006090Global Health Research. The views expressed are those of the authors and not necessarily those of Wellcome, the NIHR or the Department of Health and Social Care.

## CRediT authorship contribution statement

**Alexandra Juhász:** Data curation, Investigation, Writing – original draft, Writing – review & editing. **Mtumweni Ali:** Methodology, Validation, Writing – review & editing. **Sam Jones:** Data curation, Formal analysis, Methodology, Writing – review & editing. **Carmen Ngo:** Data curation, Formal analysis, Methodology, Writing – review & editing. **Paula Cambero Guerra:** Data curation, Formal analysis, Methodology, Visualization, Writing – review & editing. **E. James LaCourse:** Data curation, Investigation, Methodology, Supervision, Visualization, Writing – review & editing. **Othman Juma:** Project administration, Supervision, Validation, Writing – review & editing. **Shaali M. Ame:** Conceptualization, Data curation, Investigation, Methodology, Project administration, Supervision, Writing – review & editing. **J. Russell Stothard:** Conceptualization, Data curation, Formal analysis, Funding acquisition, Investigation, Methodology, Project administration, Supervision, Validation, Visualization, Writing – original draft, Writing – review & editing.

## Conflict of interest and Authorship conformation form

All authors have participated in (a) conception and design, or analysis and interpretation of the data; (b) drafting the article or revising it critically for important intellectual content; and (c) approval of the final version.

This manuscript has not been submitted to, nor is under review at, another journal or other publishing venue.
